# Crystal structure of 2-amino-*N*-(2-fluoro­phen­yl)-4,5,6,7-tetra­hydro-1-benzo­thio­phene-3-carboxamide

**DOI:** 10.1107/S2056989015018022

**Published:** 2015-10-03

**Authors:** K. Chandra Kumar, V. Umesh, T. K. Madhura, B. M. Rajesh, Chandra

**Affiliations:** a Department of Engineering Physics, HKBK College of Engineering, Bengaluru 560 045, India; b Department of Physics, JSS College for Women (Autonomous), Saraswathipuram, Mysuru 570 009, India; c Department of Physics, Atria Institute of Technology, Bengaluru 560 024, India; d Department of Studies in Physics, Manasagangotri, University of Mysore, Mysore 570 006, India

**Keywords:** crystal structure, benzo­thio­phene derivative, biological properties, hydrogen bonding

## Abstract

In the title compound, C_15_H_15_FN_2_OS, the dihedral angle between the planes of the benzo­thio­phene ring system and the fluoro­benzene ring is 3.74 (14)°. The six-membered ring of the benzo­thio­phene moiety adopts a half-chair conformation. The mol­ecular conformation is consolidated by intra­molecular N—H⋯F and N—H⋯O hydrogen bonds. In the crystal, mol­ecules are linked by N—H⋯O hydrogen bonds, generating *C*(6) [001] chains.

## Related literature   

For background to thio­phene derivatives, see: Bonini *et al.* (2005 ▸); Brault *et al.* (2005 ▸); Isloor *et al.* (2010 ▸). For inter­molecular inter­actions involving F atoms, see: Choudhury *et al.* (2004 ▸).
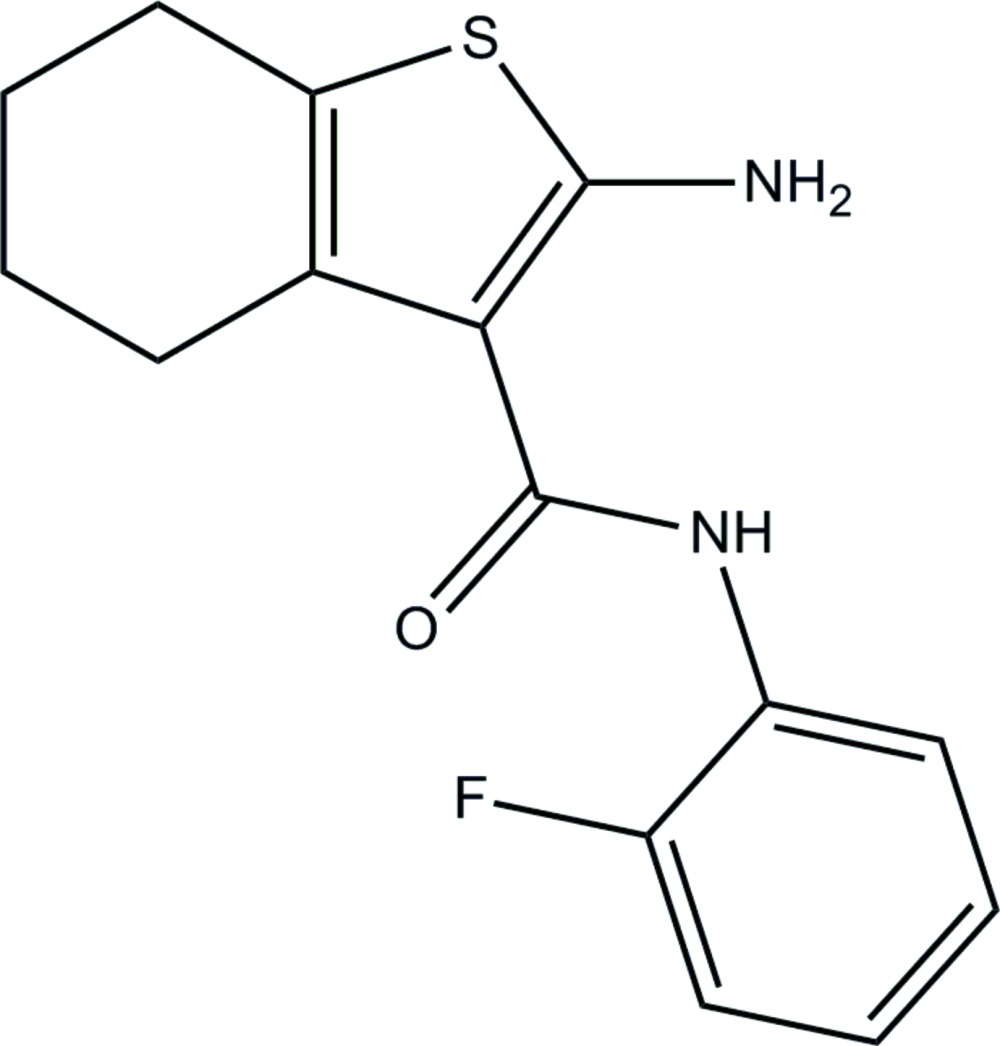


## Experimental   

### Crystal data  

C_15_H_15_FN_2_OS
*M* *_r_* = 290.36
Monoclinic 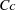

*a* = 11.213 (13) Å
*b* = 14.231 (17) Å
*c* = 9.582 (15) Å
β = 116.76 (3)°
*V* = 1365 (3) Å^3^
*Z* = 4
Mo *K*α radiation
μ = 0.25 mm^−1^
*T* = 293 K
0.30 × 0.25 × 0.20 mm

### Data collection  

Bruker APEXII CCD area-detector diffractometer
5264 measured reflections
2577 independent reflections
2363 reflections with *I* > 2σ(*I*)
*R*_int_ = 0.029

### Refinement  

*R*[*F* ^2^ > 2σ(*F* ^2^)] = 0.038
*wR*(*F* ^2^) = 0.081
*S* = 1.84
2577 reflections
182 parameters
2 restraints
H-atom parameters constrained
Δρ_max_ = 0.20 e Å^−3^
Δρ_min_ = −0.29 e Å^−3^
Absolute structure: Flack (1983 ▸)
Absolute structure parameter: 0.06 (7)

### 

Data collection: *APEX2* (Bruker, 2009 ▸); cell refinement: *SAINT* (Bruker, 2009 ▸); data reduction: *SAINT*; program(s) used to solve structure: *SHELXS97* (Sheldrick, 2008 ▸); program(s) used to refine structure: *SHELXL97* (Sheldrick, 2008 ▸); molecular graphics: *PLATON* (Spek, 2009 ▸); software used to prepare material for publication: *SHELXL97*.

## Supplementary Material

Crystal structure: contains datablock(s) global, I. DOI: 10.1107/S2056989015018022/hb7493sup1.cif

Structure factors: contains datablock(s) I. DOI: 10.1107/S2056989015018022/hb7493Isup2.hkl

Supporting information file. DOI: 10.1107/S2056989015018022/hb7493Isup3.cml

**Fig. 1.**
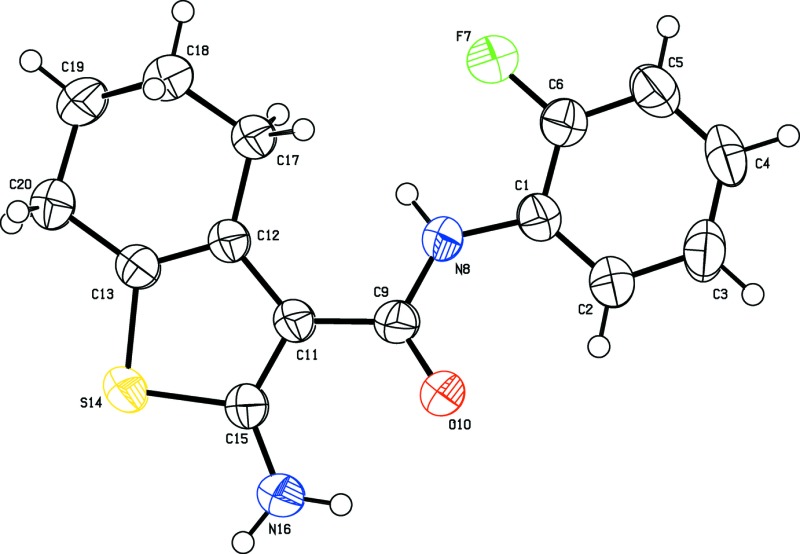
Perspective diagram of the molecule with 50% probability displacement ellipsoids.

. DOI: 10.1107/S2056989015018022/hb7493fig1.tif
Perspective diagram of the mol­ecule with 50% probability displacement ellipsoids.

**Fig. 2.**
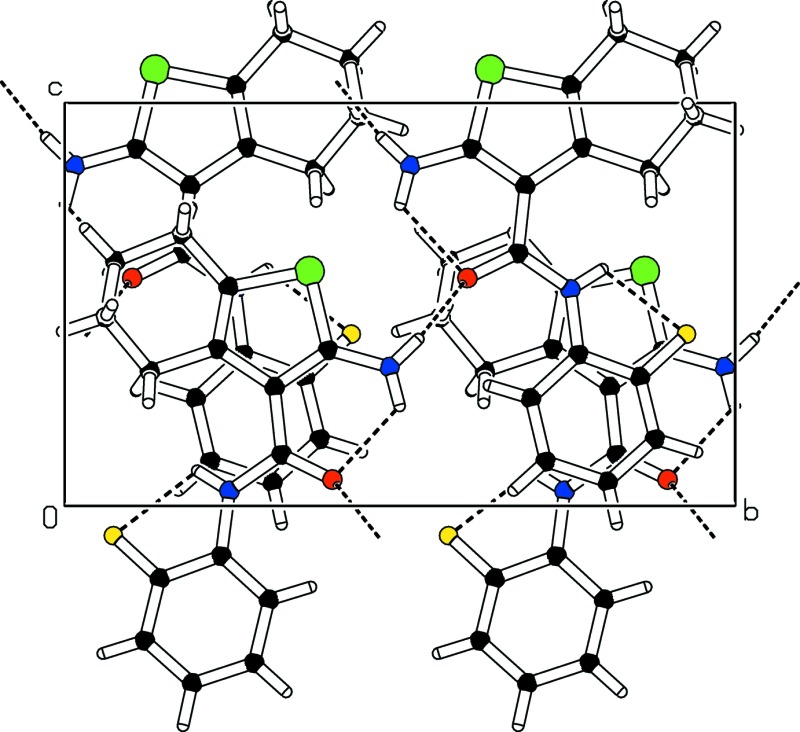
Packing diagram of the molecule viewed down the 'a' axis.

. DOI: 10.1107/S2056989015018022/hb7493fig2.tif
Packing diagram of the mol­ecule viewed down the ’a′ axis.

CCDC reference: 1045467

Additional supporting information:  crystallographic information; 3D view; checkCIF report

## Figures and Tables

**Table 1:** Hydrogen-bond geometry (, )

*D*H*A*	*D*H	H*A*	*D* *A*	*D*H*A*
N8H9*A*F7	0.86	2.26	2.643(5)	107
N16H15*C*O10	0.86	2.16	2.733(5)	124
N16H15*D*O10^i^	0.86	2.25	2.986(6)	143

Symmetry code: (i) 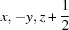.

## supplementary crystallographic information

### S1. Comment

Thiophene nucleus has been established as a potential entity in the largely growing chemical world of heterocyclic compounds possessing promising pharmacological characteristics such as anti-HIV PR inhibitors (Bonini *et al.*, 2005) and anti-breast cancer (Brault *et al.*, 2005) activities. Particularly, benzothiophene derivative shows significant antimicrobial and anti- inflammatory activities (Isloora *et al.*, 2010). In addition structures containing fluorine atoms plays a major role in intermolecular interactions (Choudhury *et al.*, 2004). The title compound was prepared and characterized by single-crystal X-ray diffraction studies.

In the molecular structure of the title compound (Fig. 1), the dihedral angle between the flurobenzene (C1–C2–C3–C4–C5–C6) and benzothiophene (C11–C12–C13–S14–C15–C17–C18–C19–C20) ring is 3.74 (14)°. The benzothiophene moiety adopts a half chair conformation conformation with puckering parameter Q = 0.475 (3) Å and φ = 215.4 (5)°, and the maximum deviation found on the puckered atom at C18 is 0.372 (4) Å. The carboximidamide unit is in anti-periplanar conformation with respect to the benzothiophene moiety, as indicated by the torsion angle value of 161.9 (3)° (N8–C9–C11–C15). The crystal structure features intermolecular N—H···O hydrogen bonds. The packing diagram of the molecule viewed down the *a* axis as shown in Fig. 2.

### S2. Experimental

Cyclohexanone (1 equiv.), 2-cyano-*N*-(2-fluorophenyl) acetamide (1.1 equiv.), elemental sulfur (1.2 equiv.), diethylamine (0.8 equiv.) was taken in ethanol and mixed thoroughly in a microwave tube. The tube was sealed and irradiated at 325 K for 15 min. After cooling ethyl acetate was added to the reaction mixture and solid residue was removed by filtration. The filtrate was concentrated under reduced pressure and purified by column chromatography to obtain yellow block shaped crystals.

### S3. Refinement

H atoms were placed at idealized positions and allowed to ride on their parent atoms with N–H distance is equal to 0.86 and C–H distances in the range of 0.93 to 0.97 Å; *U*_iso_(H) = 1.2–1.5*U*_eq_(carrier atom) for all H atoms.

### Crystal data

**Table:**

C_15_H_15_FN_2_OS	*F*(000) = 608
*M**_r_* = 290.36	*D*_x_ = 1.413 Mg m^−^^3^
Monoclinic, *C**c*	Mo *K*α radiation, λ = 0.71073 Å
Hall symbol: C -2yc	Cell parameters from 2577 reflections
*a* = 11.213 (13) Å	θ = 2.5–26.4°
*b* = 14.231 (17) Å	µ = 0.25 mm^−^^1^
*c* = 9.582 (15) Å	*T* = 293 K
β = 116.76 (3)°	Bolck, yellow
*V* = 1365 (3) Å^3^	0.30 × 0.25 × 0.20 mm
*Z* = 4	

### Data collection

**Table:**

Bruker APEXII CCD area-detector diffractometer	*R*_int_ = 0.029
ω and φ scans	θ_max_ = 26.4°, θ_min_ = 2.5°
5264 measured reflections	*h* = −13→14
2577 independent reflections	*k* = −17→17
2363 reflections with *I* > 2σ(*I*)	*l* = −11→11

### Refinement

**Table:**

Refinement on *F*^2^	Secondary atom site location: difference Fourier map
Least-squares matrix: full	Hydrogen site location: inferred from neighbouring sites
*R*[*F*^2^ > 2σ(*F*^2^)] = 0.038	H-atom parameters constrained
*wR*(*F*^2^) = 0.081	*w* = 1/[σ^2^(*F*_o_^2^) + (0.010*P*)^2^] where *P* = (*F*_o_^2^ + 2*F*_c_^2^)/3
*S* = 1.84	(Δ/σ)_max_ < 0.001
2577 reflections	Δρ_max_ = 0.20 e Å^−^^3^
182 parameters	Δρ_min_ = −0.28 e Å^−^^3^
2 restraints	Absolute structure: Flack (1983), ??? Friedel pairs
Primary atom site location: structure-invariant direct methods	Absolute structure parameter: 0.06 (7)

### Special details

**Table:**

Geometry. Bond distances, angles *etc*. have been calculated using the rounded fractional coordinates. All su's are estimated from the variances of the (full) variance-covariance matrix. The cell e.s.d.'s are taken into account in the estimation of distances, angles and torsion angles
Refinement. Refinement on *F*^2^ for ALL reflections except those flagged by the user for potential systematic errors. Weighted *R*-factors *wR* and all goodnesses of fit *S* are based on *F*^2^, conventional *R*-factors *R* are based on *F*, with *F* set to zero for negative *F*^2^. The observed criterion of *F*^2^ > σ(*F*^2^) is used only for calculating -*R*-factor-obs *etc*. and is not relevant to the choice of reflections for refinement. *R*-factors based on *F*^2^ are statistically about twice as large as those based on *F*, and *R*-factors based on ALL data will be even larger.

### Fractional atomic coordinates and isotropic or equivalent isotropic displacement parameters (Å^2^)

**Table:**

	*x*	*y*	*z*	*U*_iso_*/*U*_eq_	
S14	0.42139 (7)	0.13494 (4)	1.08277 (7)	0.0586 (3)	
F7	0.17969 (19)	0.42740 (10)	0.4264 (2)	0.0746 (6)	
O10	0.2917 (2)	0.10081 (11)	0.56571 (19)	0.0568 (7)	
N8	0.2299 (2)	0.25423 (14)	0.5376 (2)	0.0503 (7)	
N16	0.3503 (2)	0.01559 (15)	0.8447 (3)	0.0674 (9)	
C1	0.1812 (2)	0.26650 (18)	0.3762 (3)	0.0466 (9)	
C2	0.1530 (3)	0.1953 (2)	0.2671 (3)	0.0588 (10)	
C3	0.1087 (4)	0.2175 (3)	0.1106 (3)	0.0727 (11)	
C4	0.0865 (3)	0.3097 (3)	0.0601 (4)	0.0735 (13)	
C5	0.1109 (3)	0.3807 (2)	0.1661 (3)	0.0643 (11)	
C6	0.1566 (3)	0.35800 (18)	0.3201 (3)	0.0519 (9)	
C9	0.2880 (3)	0.17625 (17)	0.6279 (3)	0.0444 (9)	
C11	0.3397 (2)	0.18699 (17)	0.7955 (3)	0.0423 (8)	
C12	0.3705 (3)	0.27223 (17)	0.8901 (3)	0.0422 (8)	
C13	0.4142 (3)	0.25543 (17)	1.0436 (3)	0.0478 (8)	
C15	0.3637 (3)	0.10698 (17)	0.8869 (3)	0.0483 (9)	
C17	0.3638 (3)	0.37266 (16)	0.8342 (3)	0.0497 (9)	
C18	0.4470 (3)	0.43925 (17)	0.9684 (3)	0.0575 (10)	
C19	0.4211 (4)	0.42420 (18)	1.1078 (3)	0.0665 (11)	
C20	0.4583 (3)	0.32519 (19)	1.1743 (3)	0.0583 (10)	
H2A	0.16370	0.13280	0.29880	0.0710*	
H3A	0.09370	0.16960	0.03860	0.0870*	
H4A	0.05520	0.32350	−0.04550	0.0880*	
H5A	0.09670	0.44300	0.13370	0.0770*	
H9A	0.22220	0.30220	0.58740	0.0600*	
H15C	0.32330	0.00050	0.74820	0.0810*	
H15D	0.36900	−0.02760	0.91420	0.0810*	
H18A	0.55430	0.32090	1.23760	0.0700*	
H18B	0.41570	0.31140	1.24040	0.0700*	
H20A	0.32720	0.43510	1.07740	0.0800*	
H20B	0.47240	0.46950	1.18820	0.0800*	
H21A	0.54110	0.42920	0.99880	0.0690*	
H21B	0.42570	0.50370	0.93290	0.0690*	
H22A	0.27150	0.39340	0.78560	0.0600*	
H22B	0.39610	0.37500	0.75590	0.0600*	

### Atomic displacement parameters (Å^2^)

**Table:**

	*U*^11^	*U*^22^	*U*^33^	*U*^12^	*U*^13^	*U*^23^
S14	0.0914 (6)	0.0458 (4)	0.0421 (4)	0.0089 (4)	0.0332 (4)	0.0085 (3)
F7	0.1052 (14)	0.0470 (9)	0.0552 (10)	−0.0004 (8)	0.0217 (9)	−0.0020 (7)
O10	0.0853 (14)	0.0416 (10)	0.0432 (10)	0.0028 (9)	0.0286 (10)	−0.0026 (8)
N8	0.0726 (15)	0.0413 (11)	0.0362 (11)	0.0066 (10)	0.0239 (11)	−0.0015 (8)
N16	0.116 (2)	0.0397 (13)	0.0496 (13)	0.0007 (12)	0.0400 (13)	0.0038 (10)
C1	0.0467 (15)	0.0481 (16)	0.0397 (15)	0.0014 (11)	0.0147 (13)	0.0030 (11)
C2	0.073 (2)	0.0556 (17)	0.0419 (15)	0.0064 (14)	0.0206 (15)	0.0020 (12)
C3	0.087 (2)	0.080 (2)	0.0370 (15)	0.0112 (17)	0.0154 (15)	−0.0070 (15)
C4	0.095 (3)	0.083 (2)	0.0342 (15)	0.0095 (19)	0.0217 (16)	0.0113 (14)
C5	0.070 (2)	0.0614 (18)	0.0486 (18)	0.0004 (14)	0.0153 (15)	0.0137 (13)
C6	0.0546 (17)	0.0487 (16)	0.0459 (16)	−0.0034 (12)	0.0170 (13)	−0.0001 (12)
C9	0.0539 (17)	0.0385 (13)	0.0445 (14)	−0.0016 (12)	0.0255 (13)	0.0000 (11)
C11	0.0541 (17)	0.0391 (13)	0.0377 (13)	0.0027 (11)	0.0241 (13)	0.0029 (10)
C12	0.0534 (16)	0.0389 (13)	0.0379 (15)	0.0012 (11)	0.0238 (13)	0.0015 (10)
C13	0.0616 (16)	0.0414 (13)	0.0426 (15)	0.0068 (12)	0.0255 (14)	0.0037 (10)
C15	0.0671 (19)	0.0425 (14)	0.0408 (15)	0.0012 (12)	0.0293 (14)	0.0005 (11)
C17	0.0697 (17)	0.0404 (14)	0.0431 (13)	0.0014 (12)	0.0291 (12)	0.0014 (10)
C18	0.075 (2)	0.0433 (14)	0.0542 (16)	−0.0033 (13)	0.0292 (15)	−0.0045 (12)
C19	0.100 (2)	0.0474 (15)	0.059 (2)	0.0027 (16)	0.0418 (19)	−0.0082 (14)
C20	0.079 (2)	0.0536 (16)	0.0434 (16)	0.0055 (14)	0.0286 (15)	−0.0018 (12)

### Geometric parameters (Å, º)

**Table:**

S14—C13	1.749 (4)	C12—C17	1.516 (4)
S14—C15	1.734 (4)	C12—C13	1.346 (4)
F7—C6	1.357 (4)	C13—C20	1.497 (4)
O10—C9	1.238 (4)	C17—C18	1.530 (4)
N8—C1	1.400 (4)	C18—C19	1.505 (5)
N8—C9	1.377 (4)	C19—C20	1.525 (4)
N16—C15	1.350 (4)	C2—H2A	0.9300
N8—H9A	0.8600	C3—H3A	0.9300
N16—H15C	0.8600	C4—H4A	0.9300
N16—H15D	0.8600	C5—H5A	0.9300
C1—C6	1.388 (4)	C17—H22A	0.9700
C1—C2	1.386 (4)	C17—H22B	0.9700
C2—C3	1.387 (4)	C18—H21A	0.9700
C3—C4	1.382 (6)	C18—H21B	0.9700
C4—C5	1.370 (5)	C19—H20A	0.9700
C5—C6	1.365 (4)	C19—H20B	0.9700
C9—C11	1.449 (4)	C20—H18A	0.9700
C11—C12	1.460 (4)	C20—H18B	0.9700
C11—C15	1.387 (4)		
			
C13—S14—C15	91.95 (12)	C12—C17—C18	111.9 (2)
C1—N8—C9	129.3 (2)	C17—C18—C19	111.7 (3)
C9—N8—H9A	115.00	C18—C19—C20	112.1 (3)
C1—N8—H9A	115.00	C13—C20—C19	109.8 (2)
H15C—N16—H15D	120.00	C1—C2—H2A	120.00
C15—N16—H15D	120.00	C3—C2—H2A	120.00
C15—N16—H15C	120.00	C2—C3—H3A	119.00
C2—C1—C6	117.1 (2)	C4—C3—H3A	120.00
N8—C1—C2	125.8 (2)	C3—C4—H4A	120.00
N8—C1—C6	117.1 (2)	C5—C4—H4A	120.00
C1—C2—C3	119.9 (3)	C4—C5—H5A	121.00
C2—C3—C4	121.0 (3)	C6—C5—H5A	121.00
C3—C4—C5	119.8 (3)	C12—C17—H22A	109.00
C4—C5—C6	118.6 (3)	C12—C17—H22B	109.00
F7—C6—C5	119.4 (2)	C18—C17—H22A	109.00
C1—C6—C5	123.6 (2)	C18—C17—H22B	109.00
F7—C6—C1	117.0 (2)	H22A—C17—H22B	108.00
N8—C9—C11	116.8 (2)	C17—C18—H21A	109.00
O10—C9—N8	120.4 (2)	C17—C18—H21B	109.00
O10—C9—C11	122.8 (2)	C19—C18—H21A	109.00
C9—C11—C12	129.8 (2)	C19—C18—H21B	109.00
C9—C11—C15	118.7 (2)	H21A—C18—H21B	108.00
C12—C11—C15	111.5 (2)	C18—C19—H20A	109.00
C11—C12—C13	113.5 (2)	C18—C19—H20B	109.00
C13—C12—C17	119.3 (2)	C20—C19—H20A	109.00
C11—C12—C17	127.1 (2)	C20—C19—H20B	109.00
S14—C13—C20	120.30 (19)	H20A—C19—H20B	108.00
C12—C13—C20	128.1 (2)	C13—C20—H18A	110.00
S14—C13—C12	111.56 (19)	C13—C20—H18B	110.00
S14—C15—C11	111.52 (19)	C19—C20—H18A	110.00
N16—C15—C11	129.6 (2)	C19—C20—H18B	110.00
S14—C15—N16	118.8 (2)	H18A—C20—H18B	108.00
			
C15—S14—C13—C12	−0.1 (3)	N8—C9—C11—C12	−16.9 (5)
C15—S14—C13—C20	−178.1 (3)	N8—C9—C11—C15	161.9 (3)
C13—S14—C15—N16	178.9 (3)	C9—C11—C12—C13	178.4 (3)
C13—S14—C15—C11	−0.1 (3)	C9—C11—C12—C17	−4.5 (6)
C9—N8—C1—C2	16.1 (5)	C15—C11—C12—C13	−0.4 (4)
C9—N8—C1—C6	−165.1 (3)	C15—C11—C12—C17	176.8 (3)
C1—N8—C9—O10	−7.0 (5)	C9—C11—C15—S14	−178.6 (2)
C1—N8—C9—C11	174.7 (3)	C9—C11—C15—N16	2.4 (5)
N8—C1—C2—C3	−178.1 (3)	C12—C11—C15—S14	0.3 (4)
C6—C1—C2—C3	3.2 (5)	C12—C11—C15—N16	−178.6 (3)
N8—C1—C6—F7	−1.1 (4)	C11—C12—C13—S14	0.3 (4)
N8—C1—C6—C5	179.0 (3)	C11—C12—C13—C20	178.1 (3)
C2—C1—C6—F7	177.7 (3)	C17—C12—C13—S14	−177.1 (3)
C2—C1—C6—C5	−2.1 (5)	C17—C12—C13—C20	0.7 (6)
C1—C2—C3—C4	−2.8 (6)	C11—C12—C17—C18	−160.2 (3)
C2—C3—C4—C5	1.3 (6)	C13—C12—C17—C18	16.8 (5)
C3—C4—C5—C6	−0.2 (6)	S14—C13—C20—C19	−170.8 (3)
C4—C5—C6—F7	−179.2 (3)	C12—C13—C20—C19	11.5 (5)
C4—C5—C6—C1	0.6 (6)	C12—C17—C18—C19	−47.1 (4)
O10—C9—C11—C12	164.9 (3)	C17—C18—C19—C20	61.4 (4)
O10—C9—C11—C15	−16.4 (5)	C18—C19—C20—C13	−41.6 (4)

### Hydrogen-bond geometry (Å, º)

**Table:**

*D*—H···*A*	*D*—H	H···*A*	*D*···*A*	*D*—H···*A*
N8—H9*A*···F7	0.86	2.26	2.643 (5)	107
N16—H15*C*···O10	0.86	2.16	2.733 (5)	124
N16—H15*D*···O10^i^	0.86	2.25	2.986 (6)	143

Symmetry code: (i) *x*, −*y*, *z*+1/2.
