# Supplementary figures and images for: Crystal structure of 2-amino-N-(2-fluoro­phen­yl)-4,5,6,7-tetra­hydro-1-benzo­thio­phene-3-carboxamide
Source: Acta Crystallogr E Crystallogr Commun. 2015 Oct 3;71(Pt 11):o807–8. doi: 10.1107/S2056989015018022 (PMC4645083; doi:10.1107/S2056989015018022)

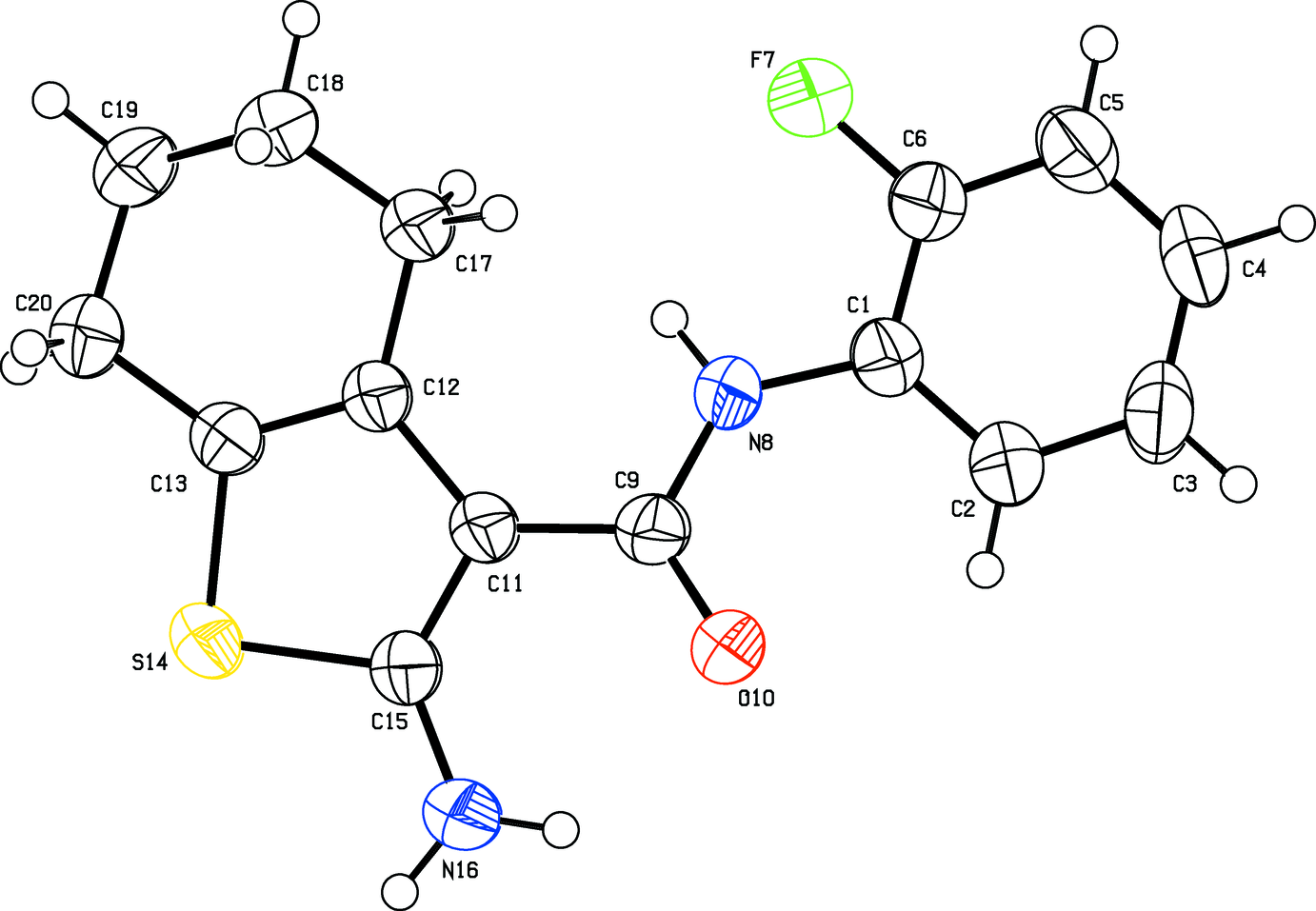

Supplement: Supplementary file 4 [file e-71-0o807-fig1.tif]

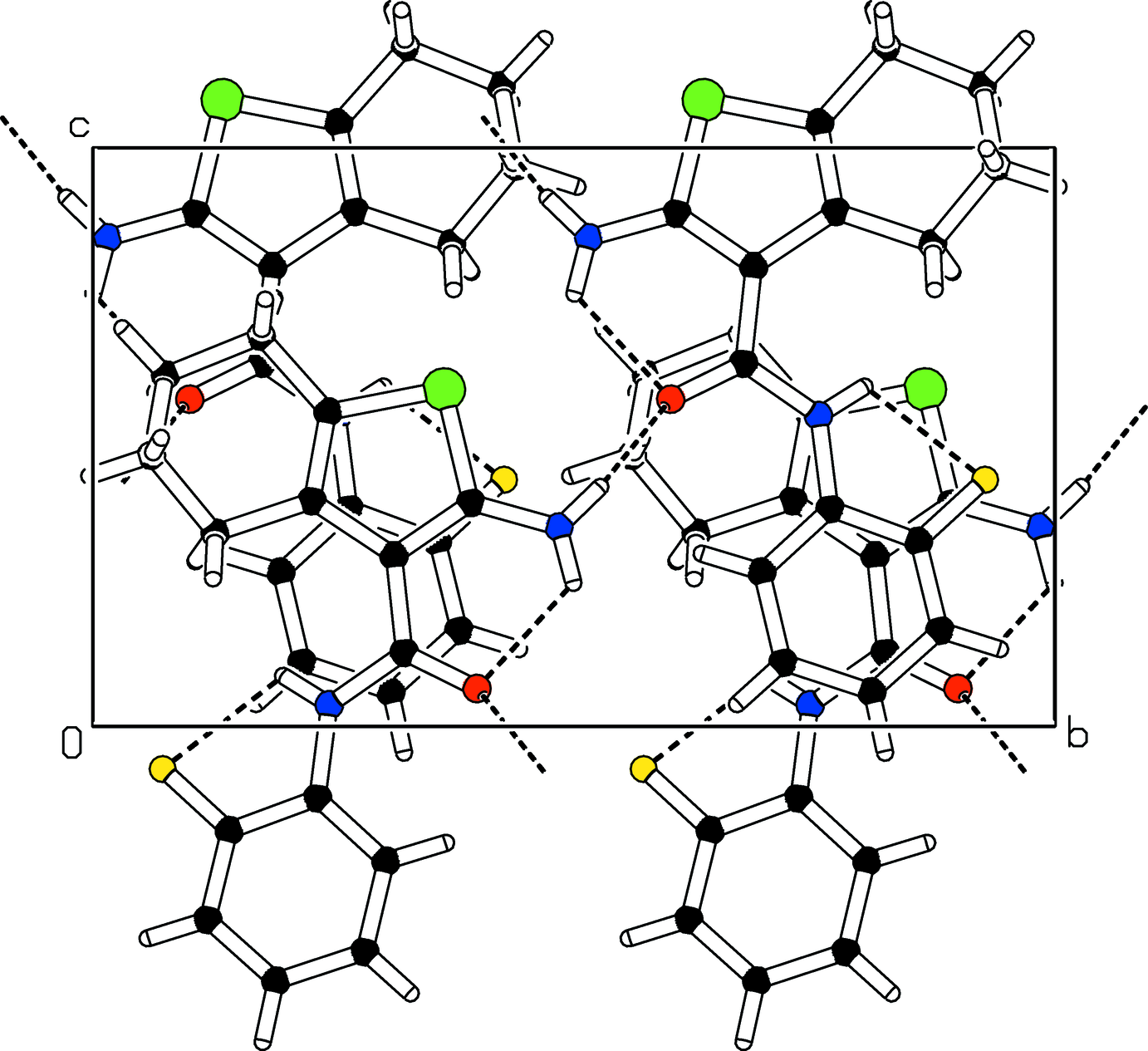

Supplement: Supplementary file 5 [file e-71-0o807-fig2.tif]
